# Unlocking Grass Stress Resistance: Fungal Endophyte-Mediated Pathogen Recognition and RNA Regulation

**DOI:** 10.3390/ijms27093899

**Published:** 2026-04-27

**Authors:** Ayaz Ahmad, Mian Muhammad Ahmed, Aadab Akhtar, Wanwan Liu, Rui Yang, Xu Sun, Xiaobin Wang, Sadia Bibi, Muhammad Bilal Khan, Shuihong Chen

**Affiliations:** 1 College of Life Science and Technology, Tarim University/State Key Laboratory Incubation Base for Conservation and Utilization of Bio-Resource in Tarim Basin, Alar 843300, China; ayazahmad3222@gmail.com (A.A.); aadabakhtar3@gmail.com (A.A.); 19882954026@163.com (W.L.); 18097511044@163.com (R.Y.); shihao0880@foxmail.com (X.S.); 17860652189@163.com (X.W.); sadiasadia6444@gmail.com (S.B.); 2 Soil and Water Science Department, Indian River Research and Education Center, Institute of Food and Agricultural Sciences, University of Florida, Fort Pierce, FL 34945, USA; mb.khan@ufl.edu

**Keywords:** fungal endophytes, abiotic stress, biotic stress, plant–microbe interactions, rhizosphere microbiome, stress signaling, multi-omics

## Abstract

Fungal endophytes are symbiotic microorganisms that establish strong relationships inside plant tissues, providing potential advantages, especially in grasses, by enhancing tolerance to both abiotic and biotic stresses. This review investigates the molecular mechanisms through which fungal endophytes mediate stress tolerance, targeting host–pathogen interactions. By modulating pathogen-associated molecular patterns (PAMPs), damage-associated molecular patterns (DAMPs), and effector proteins, fungal endophytes may contribute to priming the plant’s immune system, enhancing its resistance to pathogen invasion. Moreover, endophyte colonization regulates core processes such as osmotic regulation, reactive oxygen species (ROS) detoxification, and secondary metabolite biosynthesis that enable plants to tolerate environmental stresses like drought, heat, and salinity. The review highlights the impact of endophytes on immune priming, systemic acquired resistance (SAR), and the regulation of non-coding RNAs that regulate host gene networks associated with stress tolerance. Furthermore, the integration of advanced multi-omics techniques genomics, transcriptomics, proteomics, metabolomics, and fluxomics has revealed emerging insights into the genetic and metabolic pathways driving these symbiotic associations. However, grass-specific molecular datasets remain limited, and the consistency of endophyte-mediated tolerance across host species and environmental conditions is not yet fully resolved. Fungal endophytes increase grass stress resilience through coordinated pathogen recognition, RNA regulation, and metabolic reprogramming while AI-assisted multi-omics approaches are emerging as tools for identifying candidate regulatory networks, although empirical validation in grass–endophyte systems remains limited. Together, these advances highlight the potential for climate-smart and sustainable crop improvement. Future research integrating functional genomics, field validation, and biosafety assessment will be essential for translating endophyte-based strategies into reliable agricultural applications.

## 1. Introduction

Fungal endophytes are microorganisms that form symbiotic relationships with plant tissues without being harmful and serve as a key determinant of plant tolerance to both environmental and pathogenic stresses. Endophytic fungi colonize the internal tissues of plants, particularly grasses, without causing any disease. These fungi play an important role in host plant health and are also beneficial in helping the plants to endure adverse environmental challenges [1]. In recent years, the role of fungal endophytes has become a topic of great attention, especially with regard to climate change and growing environmental pressures affecting crop systems. Endophytes have been reported to improve tolerance to stresses such as drought, heat, salinity, oxidative damage, and pathogen invasion, although responses vary depending on host genotype, environmental conditions, and endophyte strain, making endophytic fungi a valuable resource in sustainable agriculture and management [2]. Experimental research studies in endophyte-associated grasses have demonstrated enhanced drought performance through improved leaf water status and biomass retention, increased salinity tolerance through ion homeostasis and antioxidant defense, and reduced disease severity against fungal pathogens under controlled inoculation conditions [3]. Fungal endophytes mediate stress tolerance through multifaceted interactions involving physiological modulation, metabolic reprogramming, and coordinated molecular signaling between the symbiotic partners [3]. These mechanisms are complex and involve coordinated modifications across multiple levels of biological organization, including molecular (genetic and RNA-mediated regulation) and cellular processes, metabolic pathways, and epigenetic regulation. Endophytes have the potential to improve the capacity of a plant to sustain cellular hydration, eliminate ROS, and strengthen cell walls during stress by modifying host plant physiology [4]. Moreover, they improve nutrient uptake, control phytohormonal signaling pathways, and optimize host immune systems, all of which help to boost the overall resilience of plants to stress. Fungal endophytes are increasingly explored as potential biological tools for mitigating climate-related stresses, especially in grass species that are becoming increasingly exposed to both abiotic and biotic stress [5]. However, the wider use of endophyte-mediated stress tolerance is limited in a number of ways. Endophyte specificity limits the generalizability of positive interactions between different grass species. Moreover, environmental variability (soil type, temperature, and moisture) can strongly affect interactions between plants and endophytes and result in variable performance in a field. These considerations make it difficult to predict on a long-term basis the impacts of endophyte colonization, as well as indicate the importance of grass-specific mechanistic validation.

Artificial intelligence (AI) is emerging as a complementary analytical tool in plant stress research, particularly in integrating and analyzing complex omics data to uncover hidden stress-responsive pathways and enhance stress tolerance mechanisms, including salt tolerance. Recent developments in molecular-based approaches, including genomics, transcriptomics, proteomics, metabolomics, and fluxomics, have provided insights into the molecular mechanisms of grass–endophyte symbiosis [6]. The integration of multi-omics approaches has enabled the construction of comprehensive molecular landscapes that display the genetic, regulatory, and functional networks of endophyte-induced stress tolerance. These high-throughput technologies have begun to identify candidate genes and regulatory pathways that are differentially expressed during endophyte colonization, particularly those related to stress-responsive signaling, antioxidant metabolism, and secondary metabolite biosynthesis. Transcriptomic and metabolomic analyses in endophyte-colonized grasses and cereals under drought or salt stress go through alterations in ROS-scavenging enzymes, osmoprotectant biosynthesis, phytohormone-related pathways, and defense-mediated secondary metabolites, giving experimental help for the defensive effects of endophyte symbiosis [7]. Furthermore, the elucidation of endophyte-induced metabolic reprogramming, including the accumulation of osmoprotectants, antioxidants, and secondary metabolites, has demonstrated how fungal symbionts increase the plant’s capacity to overcome oxidative damage and pathogen attack. In addition, fungal endophytes modulate the expression of stress-responsive gene regulation through non-coding RNAs, including miRNAs, siRNAs, and lncRNAs, thereby contributing to the regulatory architecture of plant–microbiome symbiosis [8]. Fungal endophyte-mediated stress tolerance involves the fine-tuned regulation of the host immune responses. Fungal endophytes influence the plant immune system through pathogen-associated molecular pattern (PAMP) recognition, damage-associated molecular pattern (DAMP) signaling, and effector-mediated immune regulation [9]. Through coordinated immune modulation, fungal endophytes may contribute to immune priming and systemic acquired resistance (SAR), which prepares the plants for a more rapid and robust response to subsequent attacks by pathogens. Moreover, endophytes produce a wide range of bioactive metabolites, including alkaloids, terpenoids, and volatile organic compounds (VOCs), which play a critical role in enhancing resistance to pathogen invasion and improving tolerance to abiotic stresses such as drought and heat. Despite these advances, knowledge gaps remain regarding the consistency of endophyte-mediated tolerance, host specificity, and grass-specific molecular validation [10].

AI–assisted integration of multi-omics datasets remains largely exploratory in grass–endophyte systems, and further experimental validation is required. This review presents the overall description of molecular principles by which fungal endophytes can contribute to stress resistance in grasses, including pathogen recognition, RNA regulation, epigenetic changes, and the coordination of multi-omics approaches. The implication of applying genomics, transcriptomics, proteomics, and metabolomics in the study of these interactions has provided knowledge that may guide future agricultural applications.

This review is based on a narrative synthesis of peer-reviewed literature on fungal endophyte-mediated stress tolerance in grasses. Relevant studies published between 2015 and 2026 were identified through searches of major scientific databases, including Web of Science, Scopus, PubMed, and Google Scholar. Search terms included combinations of keywords such as fungal endophytes, grass stress tolerance, plant–endophyte interactions, multi-omics, RNA regulation, epigenetics, immune priming, and biotic and abiotic stress. Studies were selected based on their relevance to the molecular mechanisms of endophyte-mediated stress tolerance, including multi-omics integration, RNA regulation, and immune signaling in grasses and related crop systems. Only peer-reviewed articles published in English were considered. Irrelevant or non-mechanistic studies were excluded. In total, 137 publications were used to inform the conceptual framework of this review.

## 2. Molecular Mechanisms of Fungal Endophyte-Mediated Stress Tolerance

There are various coordinated molecular mechanisms that facilitate fungal endophytes in increasing host grass tolerance to abiotic and biotic stress. The colonization of endophytes results in the regulation of host physiological processes, such as the enhancement of osmotic balance, ROS elimination, and the strengthening of cell walls. The compatibility of solutes (proline, sugars) and antioxidant enzymes (peroxidases, superoxide dismutase) in response to oxidative damage under drought and salinity stress has been demonstrated to be endophyte-associated [11]. Endophyte inoculation causes changes in stress-responsive gene expression, such as late embryogenesis abundant (LEA) proteins, heat shock proteins (HSPs), and dehydration responsive element binding proteins (DREBs), which enable plants to be better adapted to stress [12]. According to recent transcriptomic and proteomic studies, endophytes impact the phytohormone signaling networks, including abscisic acid (ABA), salicylic acid (SA), and jasmonic acid (JA), that form the core of stress perception and response [13]. These hormonal variations pre-condition host mechanisms and thereby enhance stress resistance [14]. In addition, endophytes are able to adjust host metabolic processes to increase nutrient uptake and resource distribution in stress conditions [15]. Fungal effectors and VOCs are increasingly recognized as key regulators that reprogram host gene networks involved in stress adaptation. Endophyte-associated regulation has been linked with (1) activation of antioxidant-defense genes encoding superoxide dismutase, peroxidase, and catalase; (2) modulation of phytohormone-signaling genes in the ABA, JA, and SA networks; and (3) induction of osmotic- and dehydration response genes such as DREB, LEA, and HSPs, contributing to systemic tolerance [16]. All these molecular pathways emphasize an active and complex endophyte–host interaction that enhances grass resilience to unfavorable environmental factors [17].

### 2.1. Stress Tolerance in Grasses

Fungal endophytes confer increased stress tolerance in grasses through a range of coordinated molecular mechanisms that enhance physiological and biochemical responses under harsh environmental conditions [18]. One fundamental approach by which endophytes improve stress resistance is osmoregulation. Under drought, salinity, or osmotic stress, host plants accumulate compatible solutes, osmotically active molecules such as proline and trehalose, that assist in maintaining cellular hydration, protect macromolecules, and stabilize cellular structures [19]. Endophyte-associated modulation of host metabolism promotes the levels of these osmoprotectants, which in response reduce cellular dehydration and improve water retention in stressed tissues [20]. Proline is also an osmolyte and functions as a scavenger of ROS and protein structure regulator, thereby simultaneously mitigating a wide range of stresses [21].

In addition to osmoregulation, antioxidant defenses facilitated by endophytes are critical to reduce cellular damage during stress. Abiotic stress induces excessive production of ROS, including superoxide anions, hydrogen peroxide, and the hydroxyl radical that can oxidize lipids, proteins, and nucleic acids [22]. Fungal endophytes colonize grasses that often exhibit increased activity of antioxidant enzymes and enable them to better detoxify ROS and maintain redox homeostasis [23]. Endophytes also facilitate the accumulation of non-enzymatic antioxidants, including glutathione and flavonoids, that extend the ability of the plant to withstand oxidative stress [24]. Additional key mechanisms entail the production of secondary metabolites, including alkaloids, terpenoids, and phenolic compounds, most of which provide protective benefits under stress. These compounds work as signaling molecules, antioxidants, or physical barriers, in contrast with cellular damage [25]. At the molecular level, endophyte colonization reprograms gene expression, activating stress-responsive mechanisms and promoting transcription of genes connected with water stress signaling, hormone metabolism, and cell protection [26]. Transcriptomic and proteomic studies in grasses show elevated levels of stress-related genes encoding proteins such as DREB factors, HSPs, and LEA proteins in endophyte-colonized plants under osmotic stress conditions [27]. Together, these molecular events, including osmoregulation, enhanced antioxidant defenses, secondary metabolite production, and dynamic gene regulation, underlie improved tolerance to abiotic stresses in endophyte-associated plants, with evidence derived primarily from grasses and supported by selected non-grass model systems where conserved mechanisms have been reported (Table 1) [28].

### 2.2. RNA Regulation

**Figure 1:**
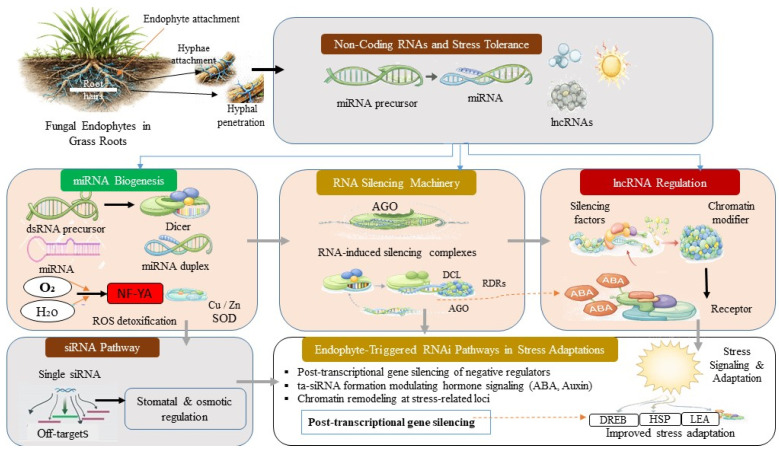
Mechanisms of RNA-mediated regulation underlying fungal-endophyte-mediated stress tolerance in grasses. The figure represents a conceptual framework summarizing proposed ncRNA-mediated regulatory pathways rather than primary quantitative data. Endophyte colonization triggers non-coding RNA regulatory pathways, including miRNA, siRNA, and lncRNA networks, that regulate RNA silencing machinery (AGO, DCL, and RDR). These mechanisms modulate phytohormonal signaling, ROS detoxification, and chromatin remodeling, leading to improved stress signal adaptation.

RNA regulation is identified as a key pathway through which fungal endophytes modulate host gene expression to increase stress tolerance in grasses. Beyond mRNAs, a complex network of non-coding RNAs (ncRNAs), including microRNAs (miRNAs), small interfering RNAs (siRNAs), and long non-coding RNAs (lncRNAs), coordinates post-transcriptional control and modulates stress signaling cascades (Figure 1) [35]. Current evidence for ncRNA-mediated regulation in grass–endophyte systems remains largely descriptive, and mechanistic validation is still limited. These regulatory RNAs may originate from both host plants and fungal endophytes, indicating cross-kingdom RNA communication. Evidence for cross-kingdom RNA transfer is primarily derived from model plant systems, with relatively few studies directly demonstrating this mechanism in grasses. Endophytes may deliver small RNAs into host cells or induce host-derived miRNAs by reprogramming transcriptional and RNA interference pathways involving Dicer-like (DCL), Argonaute (AGO), and RNA-dependent RNA polymerase (RDR) proteins. Through these mechanisms, fungal endophytes are proposed to fine-tune stress-responsive gene networks and enhance tolerance to environmental stress.

Emerging evidence suggests that cross-talk between fungal-derived small RNAs and host miRNAs could represent a novel mechanism through which endophytes mediate stress tolerance, potentially leading to a deeper understanding of fungal–host genetic interactions and their applications in enhancing stress resilience in grasses [36].

#### 2.2.1. Non-Coding RNAs and Stress Tolerance

The miRNAs are ~21–24 nucleotide RNAs that direct sequence-specific repression of target mRNAs by RNA-guided silencing complexes (RISCs). Under abiotic stress, including drought, salinity, and low-temperature stress, particular microRNAs, such as miR398, miR169, and miR156, are differentially expressed in plant systems, resulting in suppression of negative regulators of stress responses. This includes regulation of copper/zinc superoxide dismutase and NF-YA transcriptional regulators in barley, maize, and other grass species [36]. Fungal endophyte establishment has been associated with altered expression profiles of these stress-mediated miRNAs, promoting ROS detoxification and stress adaptation, including changes in miRNA levels that affect antioxidant defense mechanisms in endophyte-colonized seedlings exposed to drought [37]. The siRNAs, originated from double-stranded RNA intermediates, can silence repetitive elements or regulate the expression of stress-responsive genes. Endophytes have been reported in model experimental systems to influence biogenesis of specific siRNA populations, which are key modulators of signaling pathways such as ABA, thereby increasing drought and salinity tolerance by promoting stomatal and osmotic regulation pathways [38]. However, direct grass-specific evidence for endophyte-induced siRNA regulation remains limited. The lncRNAs (>200 nt) function as assembly platforms or decoys interacting with transcription factors, chromatin modifiers, or RNA-binding proteins; and in plant–microbe symbioses, altered expression of lncRNAs has been associated with chromatin remodeling at stress-responsive loci, permitting fast transcriptional remodeling [39].

#### 2.2.2. RNA Silencing Mechanisms

Central to ncRNA activity is the RNA silencing pathway. Fungal endophytes may influence components of the RNA interference (RNAi) pathway, including DCL proteins, AGO proteins, and RDRs in the host, mediating RNA-induced silencing complex biogenesis that guides sequence-specific cleavage or translational suppression of stress-inhibitory transcripts [40]. These regulatory components are primarily encoded by the plant genome; however, fungal endophytes can influence their expression and activity and, in some systems, may contribute to fungal-derived small RNAs that interact with host RNA silencing machinery, indicating cross-kingdom RNA regulation. At the molecular level, proposed endophyte-associated RNA silencing mechanisms include post-transcriptional gene silencing (PTGS) of negative regulators of stress adaptation, trans-acting siRNA (ta-siRNA) formation that affects hormone signaling (including ABA and auxin) to reinforce drought tolerance, and chromatin-associated RNAi, where siRNAs and lncRNAs orchestrate chromatin regulatory complex assembly at stress-responsive promoters, enhancing epigenetic accessibility and transcription [41]. These RNA-mediated regulatory pathways may interface with chromatin remodeling mechanisms, providing a functional link between ncRNA activity and epigenetic modifications described in the following section. The coordinated modulation of ncRNAs and the RNA silencing machinery provides grass with a flexible, rapid, and reversible system that helps reprogram transcriptional output in response to environmental stress, indicating that fungal endophytes may contribute to post-transcriptional regulatory networks as part of host stress resilience strategies [42].

### 2.3. Epigenetic Modifications in Host Grasses

Stable yet dynamic alterations in chromatin structure, which do not modify the underlying DNA sequence, play an important mechanistic role in grass stress responses, including adaptation following fungal endophyte colonization. These epigenetic signatures mainly involve DNA methylation, histone post-translational regulation, and chromatin restructuring, which collectively coordinate transcriptional reorganization of stress-responsive loci in the host genome [43]. Remodeling the DNA methylation landscape is one of the mechanisms through which fungal endophytes mediate host responses to stress. The presence of cytosine methylation in CG, CHG, and CHH sequence contexts can influence stress-responsive genes by modifying chromatin accessibility. Persistent DNA hypomethylation in intergenic and regulatory sequences has been associated with endophyte symbiosis, as reported in Lolium perenne under water stress conditions [44]. This hypomethylation is generally locus-specific and is thought to promote transcriptional activation of stress-responsive genes. Conversely, maintenance of dense methylation in transposable elements assists in preserving genome integrity under stress without triggering mobilization of harmful elements [45]. These seemingly contrasting patterns reflect functional partitioning of methylation, where promoter or regulatory regions may undergo hypomethylation while transposable elements remain methylated to maintain genomic stability. However, quantitative epigenomic datasets in grass–endophyte systems remain limited, and most supporting evidence outside Lolium derives from non-grass model species. 

Fungal symbionts additionally induce histone post-translational modifications, such as acetylation or methylation of histone H3 tails, which changes nucleosome stability and transcription factor access to regulatory regions of defense and pathogenesis-related genes. These modifications are mediated through endophyte-derived signals, including small RNAs, effector molecules, and secondary metabolites that modulate host chromatin-modifying enzymes such as histone acetyl transferases, deacetylases, and methyltransferases, thereby altering chromatin accessibility. These histone marks can facilitate transcriptional activation (H3K4me3 at defense genes) or repression (H3K27me3 at developmental loci), depending on the abiotic stress conditions and symbiont activation state [46]. Most evidence for these histone modifications in endophyte-associated stress responses is derived from model plant systems, with limited grass-specific validation currently available. Epigenetic regulation in endophyte-mediated grasses is coupled with gene expression networks governing immune responses and stress adaptation. Endophyte-triggered epigenetic modifications frequently co-occur with increased expression of transcription factors involved in abiotic stress signaling (including DREB, NAC, and MYB families) and hormone pathways such as ABA and SA, promoting an open chromatin state for stress tolerance [47]. Targeted mobilization of chromatin remodelers to specific gene locations, guided by DNA methylation patterns and histone codes, enables reversible shifts in transcriptional programs that allow grass species to withstand cyclical or prolonged stress. Evidence from studies of epigenetic regulation in plant–microbe interactions suggests that some epigenetic changes may be transgenerational, enabling grasses to retain stress responses modulated by symbionts and transmit primed responses to successive offspring [48]. The role of fungal endophytes in modulating epigenetic memory, particularly through DNA methylation and histone modifications, presents an exciting frontier for understanding how endophytes not only confer immediate stress tolerance but also potentially prime plants for future stress responses across generations [43]. Nevertheless, direct quantitative evidence for transgenerational epigenetic inheritance in grass–endophyte systems remains scarce. These pathways highlight how fungal endophytes affect host epigenetic architecture and gene regulation to enhance stress tolerance without altering genomic sequences, supporting epigenetic regulation as a key facet of host–endophyte interactions under environmental stress [49]. 

## 3. Pathogen Recognition and Immune System Modulation by Endophytes

Fungal endophytes play a vital role in regulating the immune system of host grasses, especially in pathogen recognition and promotion of resistance mechanisms. While important achievements have been made in understanding plant responses to biotic factors such as pathogen attack, the integration of AI tools for studying abiotic stress responses, especially salt tolerance, is an emerging field. AI offers the strength to improve data integration from different omics layers, providing insights into salt stress adaptation in a more systematic and comprehensive way. Through the combined action of PAMPs, DAMPs, and effector proteins, endophytes interact with host immunity, priming or suppressing specific mechanisms to favor plant survival under stressful conditions [50].

### 3.1. Pathogen Recognition and Signaling Networks in Grasses

**Figure 2:**
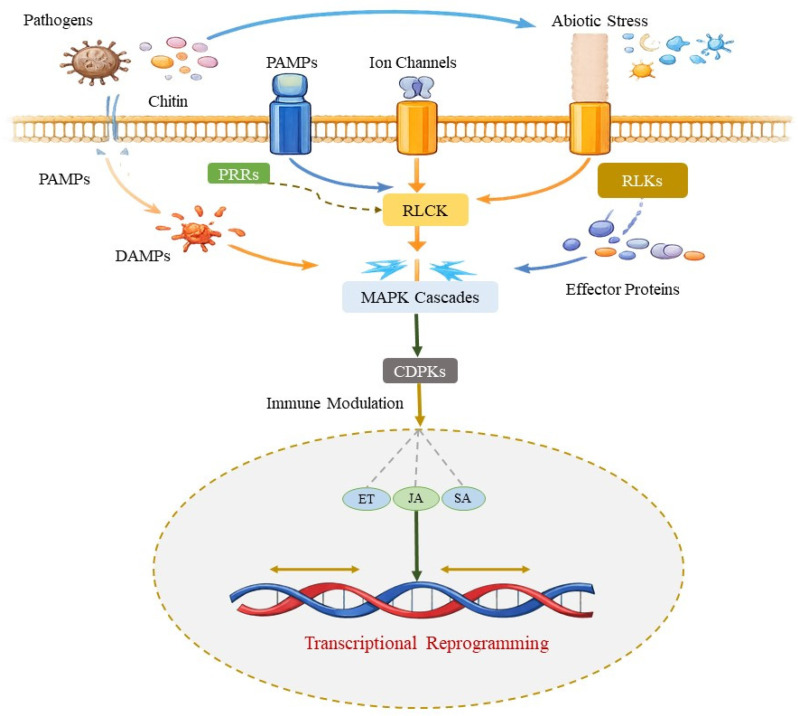
Molecular signaling networks involved in pathogen recognition and immune regulation during fungal endophyte interaction. Recognition of PAMPs and DAMPs by PRRs and receptor-like kinases (RLKs) activate receptor-like cytoplasmic kinases (RLCKs), MAPK cascades, and calcium-dependent protein kinases (CDPKs). Fungal endophytes fine-tune these pathways by attenuating excessive PTI while maintaining downstream defense competence. This controlled modulation regulates phytohormone networks, including SA, JA, and ethylene (ET), leading to balanced transcriptional reprogramming and optimized defense responses.

The initial plant defense layer involves the recognition of PAMPs, such as conserved microbial motifs (including flagellin and chitin) by pattern recognition receptors (PRRs) in the plant defense system, triggering downstream signaling cascades that regulate plant immune responses (Figure 2) [51]. In the case of fungal endophytes, endophyte-derived PAMPs can activate PAMP-triggered immunity (PTI), which is a swift, broad-spectrum immune response. Significantly, endophytes can fine-tune this defensive response through changing the expression and function of PRRs, decreasing the severity of immune reactions that would typically induce pathogenic microorganisms [52].

Below the PRR-mediated signaling, there is the activation of the MAPK and CDPK cascade that alleviates transcription factors that trigger MIR genes and other small RNA loci in the plant genome. DCL, AGO, and RDR proteins process these precursors into regulatory miRNAs and siRNAs. Moreover, fungal endophytes can also provide their own small RNAs to host cells, which suggests that regulatory RNAs are produced by plant and fungal genomes and act downstream of PAMP/DAMP signaling to control plant defense responses.

Recent studies have shown that fungal symbionts suppress excessive PTI responses in grasses, avoiding plant overreaction to harmless or beneficial microbes while retaining functional defense networks against pathogenic threats [53]. The detection of DAMPs, released from the host upon cellular damage or pathogen attack, also plays a key role in activating the plant immune system. Endophytes associate with DAMP receptors, such as receptor-like kinases (RLKs), to further refine immune responses, ensuring a balance between defense and growth [54]. Through this modulation, endophytes inhibit inappropriate defense responses that could harm the host while still activating systemic resistance pathways.

### 3.2. Effector Proteins and Immune Modulation

Endophytes release diverse effector proteins that directly interact with the host plant defensive system [55]. These effector proteins either suppress or potentiate host immune functions, allowing endophytes to avoid triggering excessive immune responses and ensure symbiotic associations. Some of these effectors resemble endogenous plant factors and cell wall compartments to facilitate immune system evasion [56]. Recent research has proposed the idea that effectors from fungal endophytes regulate plant hormonal balance, such as SA, JA, and ET, which are critical regulators of immune signaling and stress adaptation in plants [57]. By regulating phytohormonal balance, endophytes support the plant’s ability to mount defensive responses without impairing growth. Endophytes, for instance, often reduce SA levels that are frequently linked to pathogen defense, thus avoiding wasteful activation of immune pathways that may negatively affect plants under non-stress conditions [58]. Endophytes also produce volatiles and secondary metabolites (including alkaloids, terpenoids) that influence host immunity. These compounds not only provide defensive mechanisms against pathogens but also regulate the plant’s ability to withstand abiotic stress, such as drought, heat, salinity, and oxidative damage [59].

### 3.3. Immune Priming and Systemic Resistance

Fungal symbionts activate immune priming, a process in which plant immunity becomes primed to respond more efficiently to future pathogen attacks [59]. This priming results in the activation of defense-associated genes, including those encoding pathogenesis-related (PR) proteins and other defense-related proteins, while preventing unnecessary full-scale immune activation [60]. The immune priming effect can increase extending periods, providing the plant with heightened resistance during subsequent pathogen challenges. SAR is another efficient defense mechanism promoted by endophytes. Endophyte colonization can induce SAR networks, which play an important role in the upregulation of specific transcription factors (including WRKY and MYB) involved in stress and pathogen resistance [61]. By inducing SAR, endophytes provide a broad-spectrum immune response that prepares the plant for pathogen stress attack, enhancing the host’s ability to resist different types of pathogens [59,61].

Fungal endophytes effectively modulate host–pathogen associations by fine-tuning pathogen recognition, defense responses, and systemic resistance in grasses. They utilize PAMPs and DAMPs to provoke immune responses that balance growth with pathogen defense. Moreover, secreted effector molecules assist in balancing the plant immune system to achieve optimal tolerance to stress without overactivating defense mechanisms. These advanced mechanisms enable the endophytes to protect the host plant against pathogens while also providing an effective immune priming for better responses to future stresses. This critical regulation of the immune system represents an important mechanism for increasing grass resilience to biotic stress in crop production systems [50,61].

## 4. Multi-Omics Landscapes and AI-Driven Discovery in Grass–Endophyte Symbiosis

AI-mediated multi-omics involves the integration of multiple high-throughput technologies, such as genomics, transcriptomics, and proteomics, to map the molecular architecture of complex biological systems. Multi-omics approaches have been widely adopted in the field of grass–endophyte symbiosis research to reveal the genetic, regulatory, and functional aspects of how endophytes interact with the host in response to stress and immune regulation [62]. The integration of AI with multi-omics datasets enables the identification of candidate regulatory hubs and the prioritization of stress-responsive pathways; however, most applications in grass–endophyte systems remain exploratory. AI-based models, including convolutional neural networks (CNNs) and support vector machines (SVMs), are mainly applied to classification, clustering, and feature selection rather than robust predictive modeling, thus facilitating data-driven hypothesis generation for plant tolerance to environmental stress [63]. These system-level integrative approaches surpass the limitations of individual analytical platforms.

### 4.1. Integration of Genomics, Transcriptomics, and Proteomics

Genomic sequencing serves as the foundation for understanding the knowledge of both endophyte and host grass genomes. Whole genome sequencing combined with comparative genomic analyses facilitates the discovery of gene repertoires linked with symbiosis, stress tolerance, secondary metabolite biosynthesis, and signaling mechanisms that facilitate mutualistic association [64,65]. Through comparative genomics of endophytic, pathogenic, and saprophytic fungal lineages, researchers can pinpoint genetic determinants that underpin endophytic lifestyle adaptation and stress-induced responses [66]. In host grasses, the integration of genomic resources with transcriptomic data enables the identification of regulatory motifs and gene networks responsive to endophyte colonization under stress. Endophyte presence has been shown to affect expression patterns of host genes involved in stress perception, signaling, and downstream defense pathways. Endophytes also provide insight into how symbiotic establishment remodels host transcriptomes in response to environmental challenges [67,68]. Proteomic analyses, generally utilizing mass-spectrometry-based approaches, complement genomic and transcriptomic data by detecting the mature protein products and post-translational modifications that govern biological functions [69].

Proteomic analysis of endophyte-colonized grasses identifies stress-responsive proteins, signaling components, chaperones, and defense-related enzymes that exhibit altered abundance compared to non-colonized controls [70]. These proteins often participate directly in stress mitigation, immune modulation, or metabolic adjustments that promote host resilience. For instance, proteomic analyses have identified enhanced antioxidant enzyme activity, elevated levels of PR proteins, and metabolic enzymes involved in osmotic regulation, which are not always detectable from transcriptomic data alone due to post-transcriptional regulation [69]. Moreover, combined proteomic and transcriptomic datasets allow more precise functional characterization and validation of candidate stress-responsive genes identified through genomic or transcriptomic screens. By integrating genomics, transcriptomics, and proteomics, scientists assemble a multi-layered picture of how endophytes affect host biology at the DNA → RNA → protein axis, providing deeper mechanistic insight into grass adaptation and stress tolerance [68,70].

### 4.2. Metabolomics and Fluxomics

Metabolomics and fluxomics show the biochemical situation and metabolic reprogramming that occur dynamically in response to endophyte colonization and environmental stress in grasses [71]. In contrast to transcriptomics and proteomics, which measure possible regulatory changes, metabolomics address the end result of cellular processes. Real-time physiological traits and biochemical signatures are directly involved in stress resilience. Endophyte-induced metabolic reprogramming reflects coordinated shifts in primary and secondary metabolism that support osmotic adjustment, redox homeostasis, and defense signaling during stress conditions [72].

Metabolomics uses high-resolution analytical systems, gas chromatography–mass-spectrometry (GC-MS), liquid chromatography mass-spectrometry (LC-MS), and nuclear magnetic resonance (NMR)—to measure hundreds to thousands of metabolites at once [71]. Metabolic profiling identifies key metabolite categories in colonized hosts within grass endophytes and determines the numerous stress-mitigating compounds that participate directly in stress signaling in colonized hosts [72,73]. Proteins and membranes are stabilized by stable osmoprotectants such as proline, glycine betaine, and certain sugars in dehydration and osmotic stress. Antioxidants such as ascorbate, glutathione, and phenolic compounds are employed to detoxify the reactive oxygen species generated during drought or salinity [74]. Such metabolite shifts indicate that endophytes actively redirect host metabolic pathways toward stress-protective compounds rather than growth-associated metabolism. The production of secondary metabolites in response to endophyte colonization, including alkaloids (including peramine, lolines), terpenoids, and flavonoids, serves as a chemical protective barrier. These metabolites help reduce oxidative damage and pathogen attack, while regulating the hormone signaling pathways (including ABA and JA) required to adapt to stresses [71,74]. Since metabolites are close to the phenotype, metabolomic signatures are powerful biomarkers of stress tolerance, and have been utilized to differentiate between tolerant and sensitive grass lines following endophyte association [75].

Fluxomics, the quantitative analysis of enzymatic reaction rates, provides insights into how carbon and nitrogen flow through biochemical networks in response to endophyte colonization and stress. Through stable isotope tracing (^13^C, ^15^N) combined with computational modeling, researchers can trace the distribution and partitioning of substrates within central metabolism [75]. Under drought or salt stress in endophyte-associated grasses, flux analysis shows enhanced allocation of carbon toward protective biosynthetic pathways. These pathways, such as polyamine and phenolic metabolism, are prioritized at the expense of growth-related biomass production [76]. Nitrogen flux studies reveal that fungal endophyte presence often increases amino acid synthesis and nitrogen redistribution, enhancing the biosynthesis of proteins related to stress response and antioxidants while stabilizing osmotic balance. Endophyte-mediated alterations in flux through tricarboxylic acid (TCA) cycle intermediates suggest a metabolic shift toward enhanced energy and redox balancing under stress conditions [76]. These flux changes demonstrate that endophytes modify resource allocation strategies, promoting survival-oriented metabolism under adverse environmental conditions.

Together, metabolomics and fluxomics reveal the metabolic networks that determine the physiological state of the endophyte-colonized state and reprogram primary and secondary metabolism, regulating the use of carbon and nitrogen to mitigate stress effects and enhance physiological resilience. These approaches not only identify key metabolites and pathways but also measure real-time changes in metabolism that help elucidate the host–endophyte interaction at multiple biological levels (Table 2). Therefore, metabolomic and fluxomics integration provides mechanistic insight into how endophytes translate molecular signaling into functional stress tolerance traits. They provide mechanistic insights, which are becoming significant for translating basic research into genetic improvement of grass stress tolerance [71,72]. Mediation of multi-scale metabolic flux modeling with AI-driven data analysis enables the dynamic simulation of metabolic shifts, offering a predictive framework for optimizing stress resilience mechanisms in grasses under varying environmental stress conditions [75,76].

### 4.3. AI–Omics Synergy: From Descriptive Mapping to Predictive Precision Discovery

**Figure 3:**
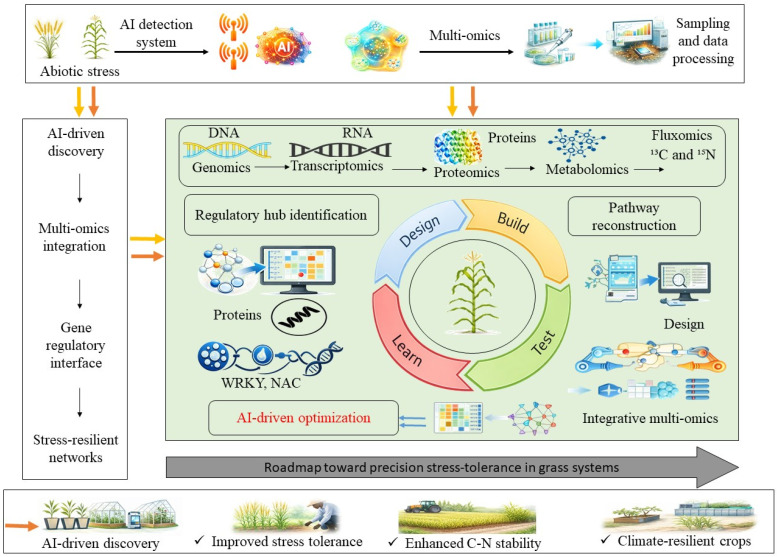
AI-driven multi-omics framework for stress tolerance discovery in grass–endophyte systems. The diagram represents a conceptual workflow illustrating integration of multi-omics datasets with AI-based analytical steps rather than a validated predictive pipeline. AI integrates genomics, transcriptomics, proteomics, metabolomics, and fluxomics (^13^C/^15^N tracing) to identify candidate regulatory pathways involved in abiotic stress. Quantitative outputs include differential gene expression, metabolite fold-change, and network-based prioritization of transcription factor modules (WRKY and NAC).

The next step involves transitioning from descriptive multi-omics, which primarily catalogs changes along the DNA–RNA regulatory axis, to data-driven hypothesis generation enabled by the integration of AI and machine learning (ML) [84]. However, AI applications in grass–endophyte systems remain limited, and predictive performance metrics are rarely reported. The integration of AI with multi-omics datasets enables identification of candidate regulators based on quantitative metrics such as differential gene expression (log_2_ fold change), metabolite abundance, and network centrality scores (Figure 3). While standard omics provides a snapshot of the symbiotic networks, AI-based architectures such as CNNs and SVMs allow researchers to unravel the intricate host–endophyte molecular dialogue [85,86]. An important innovation in this synergy is AI-assisted phenotyping. By using hyperspectral, thermal, and fluorescence imaging, CNN-based models detect pre-symptomatic physiological alterations in grasses, including minute changes in photosynthetic efficiency well before visible wilting symptoms appear [85]. The combination of AI and multi-omics not only improves our understanding of grass–endophyte interactions but also enables the identification of dynamic molecular markers that predict plant stress tolerance before visible symptoms appear, facilitating the development of early detection systems for grass health monitoring [84]. The phenotypic fingerprints are subsequently correlated with real-time multi-omics data to locate the molecular triggers of stress tolerance. For instance, long short-term memory (LSTM) networks can resolve dynamic transcriptomic changes, enabling the identification of early-warning miRNAs and transcription factor networks, such as WRKY and NAC families, that are activated in response to endophyte colonization [86].

Additionally, biostimulant development is transitioning toward a Design-Build-Test-Learn (DBTL) framework [87]. Within this framework, generative AI and structural biology techniques such as AlphaFold give template-free 3D predictions of symbiotic proteins, facilitating silico modeling of protein–protein linkage and metabolic flux. This enables the strategic design of targeted interventions, allowing scientists to determine how stabilizing a specific transcription factor complex or stimulating a key biosynthetic node (e.g., the TOR-SnRK2 system) will promote holistic resilience [88,89]. These approaches currently provide candidate targets for experimental validation rather than predictive deployment. AI–omics integration therefore functions as a complementary analytical framework for prioritizing stress-associated pathways. Further experimental validation and field-scale testing are required before predictive applications in grass–endophyte systems.

## 5. Biocontrol, Microbiome-Mediated Protection, and Sustainable Disease Management

### 5.1. Fungal Endophytes as Biocontrol Agents

Fungal endophytes have gained considerable biocontrol agents capable of suppressing plant pathogens both within host tissues and in the surrounding soil environment, thereby offering that they are harmless to the environment and alternative to unexceptional chemical pesticides [90,91]. In distinction from non-symbiotic antagonists, endophytes affect the plant tissues, making strong relationships that enhance host plant fitness while constraining the pathogen formation and proliferation. These organisms produce a wide range of bioactive secondary compounds, including antibiotics, antifungal polyketides, phenolics, and VOCs. They directly suppress the growth of pathogenic fungi, bacteria, and oomycetes by disorganizing cell walls intrusive with specific enzyme systems or changing the pathogen metabolism [92,93]. Beyond the production of antimicrobial metabolites, many endophytic fungi produce hydrolytic enzymes such as chitinases, glucanases, and proteases, which degrade pathogenic cell walls and constantly reduce invasion and colonization [94]. This enzymatic activity, together with the number of nutrients and locational competition within the plant, effectively limits pathogen spread [95]. Notably some genera *Trichoderma* and *Chaetomium* are well documented for these biocontrol systems, directly limiting diverse phytopathogens via antibiosis, mycoparasitism, and competitive exclusion [96]. Field and greenhouse studies report that endophyte inoculation can reduce disease severity by approximately 20–60% depending on host species, pathogen pressure, and environmental conditions. In grass systems, endophyte-associated plants have shown reductions in foliar disease incidence, improved biomass retention, and increased survival under pathogen stress compared with non-colonized controls. In addition, some endophytes initiate host protection mechanisms through induced systemic resistance (ISR) and SAR. Endophytes can trigger immune priming, whereby basal defense responses remain low under non-stress conditions but are rapidly activated when a true pathogen is encountered. This primed state reconciles the apparent reduction in basal defense responses with enhanced resistance, as endophytes suppress excessive immune activation while maintaining readiness for stronger and faster defense upon pathogen attack. Consequently, the host responds more rapidly with increased levels of defense enzymes, phytoalexins, and pathogenesis-related proteins [97,98]. Together, these direct antagonistic effects and measurable reductions in disease incidence demonstrate the practical biocontrol potential of fungal endophytes in integrated disease management systems. The combination of direct (antimicrobial) and indirect (defense stimulation) pathways enables endophytes to control pathogens efficiently within integrated disease management frameworks while decreasing dependence on toxic agrochemicals and supporting sustainable agricultural production [99]. Current era advancements in synthetic biology and metabolic engineering provide promising avenues for enhancing the biocontrol potential of fungal endophytes, allowing the production of tailored biocontrol agents that are more efficient and resilient under various environmental conditions.

### 5.2. Microbiome-Mediated Protection

The plant microbiome is the entire microbial assemblage that is totally linked with the plant that plays an important role in making the host healthy and disease resistance. Endophytes are an important part of this microbiome and regulate ecosystem level influences that go beyond their direct association with the host plant [100,101]. Through modulation of microbiomes, endophytes protect environments that are less hospitable to pathogens and more supportive of symbiotic microorganisms [102]. Endophytes can increase the prevalence of antimicrobial or beneficial microbes in both the phyllosphere and rhizosphere, generating a habitat that suppresses pathogen establishment via competitive exclusion, nutrient challenge, and antibiosis [103]. This effect is frequently noted when endophyte-associated plants exhibit reduced pathogen load even without direct contact between the endophyte and the pathogen, demonstrating the microbiome’s function as an intermediary defensive layer [102,104].

Moreover, endophytes affect the host immune system indirectly by promoting microbial signaling within the plant. They induce local and systemic immune responses that change the microbiome’s behavior, elevating the expression of host defensive genes and the deployment of defense molecules [105,106]. These changes not only reduce disease risk but also improve the overall tolerance of the plant microbiome against disturbances such as drought, soil depletion, or pathogen invasion. This helps demonstrate the interconnected nature of microbiome activities in host–pathogen associations [104,107]. By these special beneficial microbial partnerships, endophytes support maintaining the balance and robust microbiome that bolsters grass health and fortifies pathogen resistance under various environmental stress conditions [105]. 

### 5.3. Environmental Sustainability in Disease Management

In response to environmental limitations related to chemical pesticide overuse, environmental degradation and the emergence of fungicide-resistant pathogen strains, the use of endophytes offers an attractive path toward green disease management strategies. Endophyte-based techniques help lower chemical input requirements, reduce environmental contamination, and give protection to beneficial insects and soil biota, linking with sustainable agriculture and environmental guardianships [108]. One important benefit of endophyte-mediated disease control is its compatibility with integrated pest management (IPM) networks. These networks unify cultural interventions, resistant crop varieties, and biological agents to handle disease while protecting biodiversity. Endophytes, through their dual role in disease degradation and promotion of plant vigor, promote IPM efficacy and prolong its scope beyond pathogen control to include soil health enhancement and nutrient cycling [109]. Moreover, biocontrol using endophytes has been linked to a reduction in emissions of greenhouse gases, reduction of energy requirements, and enhanced soil carbon sequestration. It offers a multi-dimensional contribution to climate-smart agriculture compared to the conventional systems of management. With continued research, a combination of endophyte-based systems and microbiome engineering with omics-guided selection should ultimately lead to highly refined inoculants with crop- and ecosystem-specific adaptations, which will additionally lead to disease suppression and environmental sustainability. Conclusively, fungal endophytes that are dynamic and environment friendly provide a disease management solution and control pathogens through direct and indirect mechanisms, which is also helpful in supporting ultimate ecological goals, which will become a cornerstone to climate-friendly agriculture for the future [108,109]. Future integration of multi-omics approaches with endophyte-based biocontrol systems will offer new insights into the functional diversity of microbial communities. They can play a role in enhancing the resilience of agricultural systems against pathogens and environmental stressors [11].

## 6. From Lab to Land: Scaling Fungal Endophytes for Climate-Smart Agriculture

Fungal endophytes have received increasing scientific interest for their ability to enhance the tolerance of grasses and other crops facing challenges of climate-change-related stresses, such as drought, heat, salinity, and unpredictable precipitation [110]. These symbiotic fungi offer multiple ranges of benefits that promote plant growth, improve production, and elevate the resistance of grass to both abiotic and biotic stresses [111].

Endophytes support grass resilience through various mechanisms like osmotic regulation, where they promote the production of osmoprotectants, such as proline and trehalose, that facilitate the plant’s ability to maintain cellular hydration during water stress [112]. Additionally, endophytes increase antioxidant systems that reduce oxidative damage under extreme temperatures or drought. This antioxidant defense mechanism is mediated by elevating the expression of genes involved in the detoxification of ROS scavenging, thereby protecting plants from oxidative stress [113]. Furthermore, the presence of endophytes has been shown to promote root growth and depth, enhancing the plant’s ability to acquire water and nutrients in drought-prone conditions [114]. Through the enhancement of root architecture and nutrient absorption, endophytes help grasses deal well with nutrient stress and reduced water availability [115]. Endophyte colonization regulates multiple physiological traits, including chlorophyll stability, root architecture, antioxidant activity, and water-use efficiency, that conclusively promote plant stress resilience (Table 3).

**Figure 4:**
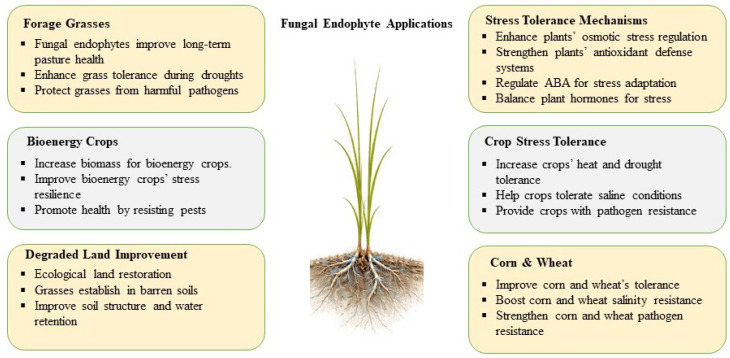
Applications of fungal endophytes in climate-smart agriculture. The diagram illustrates how fungal endophyte inoculation increases plant stress tolerance through improved osmotic regulation, antioxidant defense, and hormone balance. These mechanisms increase resistance to drought, heat, salinity, and pathogens. Endophytes that enhance the performance of forage grasses, bioenergy crops, and major crops such as corn and wheat also further assist in degraded land restoration and sustainable agriculture.

Fungal endophytes also affect the hormonal balance in the plant through the upregulation of phytohormones ABA, gibberellins, and cytokinins, which play a vital role in stress response [122]. These changes may occur either through direct production of phytohormones by endophytic fungi or through endophyte-induced regulation of host hormone biosynthesis and signaling pathways, leading to altered hormonal homeostasis during stress conditions. ABA stimulates stomatal closure, reducing water loss during drought, while the support of gibberellins and cytokinins maintain plant growth and root development under unfavorable conditions [123]. Through these mechanisms, endophytes enhance the plant’s ability to save water and optimize resource allocation under conditions of environmental stress [124]. The application of endophyte-based inoculants has been reported to be effective in improving grass production and tolerance in various stress conditions [125]. These bioinoculants are gaining widespread use in agricultural settings as part of sustainable farming practices, providing an environment-friendly alternative to chemical fertilizers and pesticides [126]. To produce endophyte-colonized plants that would be used to enhance crops in the future, there are several inoculation strategies that can be utilized. These are seed coating with fungal inoculum, root dipping of seedlings, soil drenching, and foliar application, which enable endophytes to colonize the host tissues on the systemic level [127]. Seed inoculation allows for entering symbiosis at the early stage of germination, and root inoculation allows entering the parasite by way of root tissues. Foliar treatment permits the endophyte to enter by way of the stomata or wounds. Moreover, the vertical transmission of the colonized parent plants or the engineering of the microbiome can be used to generate stable endophyte-associated crop lines [128]. The strategies offer effective systems to enhance crops with useful endophytes and stability of colonization at the field level. Endophyte-inoculated grasses exhibit increased drought tolerance, enhanced resistance to pathogens, and improved overall plant performance (Figure 4) [129]. *Epichloë* endophytes in tall fescue and other forage grasses are known to promote heat resistance and drought stress, along with increased biomass production, making them suitable for use in pasture and forage management [130].

Endophytes are being transferred to bioenergy crop systems, where they enhance the resilience of crops such as switchgrass and miscanthus under challenging environmental stress conditions [131]. These crops are key to biofuel production and are very sensitive to stress in the environment, although their productivity can be enhanced by endophyte association in the presence of unfavorable environmental factors like high temperature and scarce water supply [132]. Endophytes improve plant tolerance, stability, and sustainability of bioenergy crops under climate change conditions by stimulating their growth, biomass production, and tolerance to stresses [133]. In addition to the use of endophytes in increasing productivity, they also represent valuable resources for ecological restoration and recovery of degraded lands [134]. They have been used in reforestation projects, particularly in areas where soil degradation, desertification and poor soil water-holding capacity have been experienced. Endophyte-based inoculants can help in the preservation of ecosystems and stabilization of soil landscape areas that face difficulties due to climate, thereby enhancing the successful establishment and survival of grass species in degraded environments [135]. Overall, endophytes play a key role in climate-smart agriculture by enhancing plant resilience, reducing reliance on chemical inputs, and making agricultural systems more sustainable [136]. These advantages make endophyte-based solutions a key component in managing all the constraints of climate change, particularly in grassland ecosystems and agroecosystems, which face rising water, temperature stress, and pathogen pressure [137]. With further progress in research, the integration of fungal endophytes into agricultural and ecological systems likely provides a natural and sustainable solution to improve productivity and robustness against global climate change.

## 7. Limitations and Future Outlooks

Although fungal endophytes hold a meaningful impact on enhancing plant stress tolerance and resilience, many challenges hinder their universal application in crop production systems. One of the primary challenges is the host specificity of endophytes. It results in a substantial variation in effectiveness among plant species. This variability complicates the development of universal bioinoculants and inhibits their widespread adoption in farming systems. Furthermore, abiotic factors such as humidity, soil type, and temperature can strongly affect the survival and effectiveness of fungal endophytes. This makes it difficult to predict their field performance under different conditions. There is still limited understanding of the broad spectrum of bioactive compounds produced by endophytes and the mechanism by which these metabolites affect plant physiology and systems. The complex nature of plant–microbe interactions, combined with environmental variability, requires more research into the molecular mechanisms that produce endophyte-mediated benefits to plants. Despite these advances, the precise mechanisms underlying endophyte-mediated regulatory reprogramming, cross-kingdom signaling, and stable colonization remain incompletely understood. In particular, how endophyte-derived signals, small RNAs, and metabolic cues coordinate long-term host adaptation require further investigation. Addressing these gaps will be essential for developing reliable endophyte-based crop improvement strategies. Moreover, another challenge to the commercialization of endophyte-based solutions is the biosafety and regulatory constraints and the need for scalability in bioinoculant production.

The strategic application of innovative and advanced technologies holds great potential for mitigating these challenges and enhancing fungal-endophyte-based solutions. CRISPR-Cas9 gene editing provides an innovative approach to improving the symbiotic relationship between endophytes and plants. By using CRISPR to modify both fungal genomes and plant genes, researchers can promote host receptivity to beneficial endophytes and improve endophyte stress tolerance. They can also potentially produce engineered endophytes that are far better suited to challenging environmental stress conditions. Additionally, nanotechnology offers a viable solution for improving the execution and viability of fungal endophytes. Nanotechnology-based formulations can protect endophytes from strict environmental conditions during their application, promote their colonization efficiency in plant tissues, and enhance their persistence in soil environments. These advancements, when integrating with multi-omics technologies, will allow for greater precision in regulating stress resilience networks. They pave the way for sustainable agricultural practices that enhance crop production and climate resilience. As research continues to progress, these innovations will enable more targeted approaches for stress management, ultimately stimulating the global integration of fungal-endophyte-based bioinoculants in agriculture.

## 8. Conclusions

Fungal endophytes constitute a versatile and promising approach to improving both abiotic and biotic stress resilience in grasses and other crops. Symbiotic fungi colonize host tissue persistently as endophytes confer advantages under environmental stress conditions, including heat, salinity, drought, and pathogen attack. By reprogramming host metabolic pathways, including ROS detoxification, enhanced osmotic balance, and improved nutrient acquisition, fungal endophytes provide a biologically derived, sustainable alternative for increasing stress tolerance. Through molecular mechanisms involving transcriptional regulation, epigenetic modifications, and RNA modulation, endophytes orchestrate integrated responses that allow grasses to adapt to challenging environments. Moreover, their potential to regulate hormone-mediated responses, immune system activation, and secondary metabolite production further strengthens their role in enhancing plant defense systems. Importantly, this review integrates these molecular insights with recent advances in multi-omics approaches to provide a broader understanding of stress tolerance in grass–endophyte systems. Artificial intelligence–assisted integration of multi-omics datasets represents an emerging analytical framework; however, predictive applications in grass–endophyte systems remain limited and require experimental validation. These approaches may support the development of climate-resilient and sustainable agricultural strategies. Looking ahead, integration of multi-omics data can improve systems-level understanding of plant responses to salinity and other stresses and may assist breeding programs aimed at developing stress-tolerant crops. These insights are beneficial for the advancement of bioinoculants that can be optimized for specific crops and environmental conditions. Despite these advances, several challenges limit large-scale application of fungal endophytes. Regulatory approval requirements, biosafety assessment, and ecological risk evaluation remain major barriers for deployment, particularly when introducing non-native or engineered endophytes into agricultural systems. Potential risks, such as unintended effects on native microbiomes, horizontal gene transfer, and variability in field performance, must be carefully evaluated through long-term field trials. In addition, standardization of inoculum formulation, host specificity, and environmental stability remain critical constraints for commercialization. Although challenges such as host specificity and environmental variability persist, addressing regulatory and biosafety considerations alongside mechanistic validation will be essential for practical deployment. By fostering innovation and interdisciplinary collaboration, fungal endophytes have the potential to contribute to sustainable agriculture and improved crop resilience under changing climatic conditions.

## Figures and Tables

**Table 1:** Mechanisms of fungal endophyte-mediated stress tolerance across multiple abiotic stresses with representative examples from grasses and selected non-grass model systems where grass-specific evidence is limited.

Stress Type	Mechanistic Pathway	Endophyte Host	Measured Host Responses	References
Salt Stress	Ionic homeostasis, ROS reduction	*Ulocladium* sp., *Penicillium citrinum*	Na^+^ reduced by 40% in roots; K^+^ maintained near control levels under 150 mM NaCl	[29]
Drought Stress	Osmotic regulation, antioxidant enzyme enhancement	*Neocamarosporium* spp., *Periconia macrospinosa* (tomato & cucumber)	Increased proline, CAT, POD, SOD activities; higher chlorophyll & biomass under salt and drought	[29]
Drought Stress	Antioxidant regulation, lower oxidative injury	*Aspergillus terreus* (PP038155.1) in wild plants	Decreased MDA, electrolyte leakage; increased proline, SOD, chlorophyll under 150–300 mM NaCl and 10–20% PEG	[30]
Salt Stress	Phytohormone modulation, enhanced antioxidant defense	*Aspergillus terreus* CR7 in *Vigna radiata*	IAA production (~23 µg/mL); CAT & SOD upregulated; reduced electrolyte leakage, proline & MDA under 150–250 mM NaCl	[31]
Heat Stress	Stress metabolite regulation & ROS modulation	*Thermomyces* sp., *Aspergillus niger*	Increased antioxidant enzymes & specialized metabolites; improved growth under elevated temperature	[32]
Cold Stress	Stress gene expression & osmolyte accumulation	*Piriformospora indica* (Arabidopsis, barley)	Upregulation of cold-regulated genes; increased SOD & CAT; reduced MDA under low temperature	[32]
Alkaline Stress	Secondary metabolite production, pH buffering	*Fusarium oxysporum*	Lignin content reduced by 50%, increase in phenolic compounds by 15% under alkaline stress	[33]
Temperature + Osmotic Stress	Integrated osmotic and redox buffering	Diverse fungal endophyte taxa	Increased proline accumulation, enhanced CAT, SOD and APX antioxidant activity, improved chlorophyll content and biomass under combined stress conditions	[34]

**Table 2:** Quantitative and Mechanistic Approaches for Dissecting Endophyte–Plant Interactions.

Approach	Mechanistic Insight	Key Quantitative Metrics	Example Calculation	References
Dual RNA-seq	Simultaneous analysis of plant and fungal gene expression during stress	TPM/CPM counts; log_2_ fold change; pathway enrichment (FDR)	log_2_FC = log_2_ (TPM_stress_/TPM_control_)	[77]
^13^C isotope tracing	Quantifies carbon transfer from host plant to fungal biomass	Atom % ^13^C enrichment; carbon allocation rate	Atom% excess = sample − baseline	[78]
^13^C/^15^N labeling systems	Tracks nutrient flow between plant tissues and symbiotic microbes	δ^13^C, δ^15^N enrichment values	Isotope mixing models for nutrient partitioning	[79]
Microbiome network analysis (SPIEC-EASI)	Identifies microbial interaction networks and keystone taxa	Node degree; centrality; network edges	Graphical model inference for compositional data	[80,81]
LC–MS metabolomics	Detects metabolic reprogramming induced by endophytes	Peak intensity; metabolite fold-change; CV	Fold change = treated/control	[82]
RNA-seq + microbiome profiling	Links host transcriptional responses with microbial community shifts	Relative abundance (%); α-diversity (Shannon index)	Δ abundance = log_2_ ratio between treatments	[83]
Targeted ionomics	Reveals ion homeostasis under salt or metal stress	Ion concentrations (mg g^−1^ DW); K^+^/Na^+^ ratio	K^+^/Na^+^ = [K^+^]/[Na^+^]	[83]
Antioxidant enzyme assays	Measures oxidative stress buffering capacity	SOD, CAT activity (U mg^−1^ protein); MDA levels	% change = (treated − control)/control × 100	[83]

**Table 3:** Plant Physiological Traits Modulated by Fungal Endophyte Colonization.

Plant Trait	Endophyte-Induced Effect	Quantitative Indicators/Physiological Changes	Representative Study
Chlorophyll Content	Endophyte colonization maintains photosynthetic pigment stability under abiotic stress	12–25% increase in chlorophyll a+b and improved photosynthetic efficiency under drought and salinity stress	[116]
Water-Use Efficiency (WUE)	Improved stomatal regulation and osmotic balance	Increased WUE and reduced transpiration losses under water deficit conditions	[116]
Root Architecture	Endophytes stimulate root elongation and lateral root development	Root length and lateral root density increased by ~20–35%, improving water acquisition under drought	[117]
Nutrient Uptake (N, P, Fe)	Enhanced nutrient acquisition via improved root surface area and microbial nutrient mobilization	Increased uptake of N and P (20–40%) and improved Fe availability in endophyte-associated plants	[118]
Root Exudate Composition	Endophyte colonization alters exudate profile and amino acid secretion	Increased release of amino acids, organic acids, and sugars that influence rhizosphere nutrient cycling	[119]
Antioxidant Enzyme Activity (SOD, CAT, POD)	Activation of antioxidant defense systems to detoxify reactive oxygen species	SOD, CAT, and POD activities increased by 30–60% under drought or salt stress	[120]
Secondary Metabolite Production	Endophytes stimulate phenolics, flavonoids, and other stress-related metabolites	Elevated phenolic and flavonoid concentrations associated with improved oxidative stress tolerance	[121]

## Data Availability

No new data were created or analyzed in this study. Data sharing is not applicable to this article.

## Abbreviations

The following abbreviations are used in this manuscript:

ABA	Abscisic acid
AGO	Argonaute protein
AI	Artificial intelligence
APX	Ascorbate peroxidase
CAT	Catalase
CDPK	Calcium-dependent protein kinase
CNN	Convolutional neural network
CPM	Counts per million
CRISPR	Clustered regularly interspaced short palindromic repeats
DAMPs	Damage-associated molecular patterns
DCL	Dicer-like protein
DBTL	Design–Build–Test–Learn
DREB	Dehydration-responsive element-binding protein
DW	Dry weight
ET	Ethylene
FDR	False discovery rate
GC-MS	Gas chromatography–mass spectrometry
HSPs	Heat shock proteins
IAA	Indole-3-acetic acid
IPM	Integrated pest management
ISR	Induced systemic resistance
JA	Jasmonic acid
LC-MS	Liquid chromatography–mass spectrometry
LEA	Late embryogenesis abundant proteins
lncRNA	Long non-coding RNA
LSTM	Long short-term memory network
MAPK	Mitogen-activated protein kinase
MDA	Malondialdehyde
miRNA	MicroRNA
ML	Machine learning
ncRNA	Non-coding RNA
NMR	Nuclear magnetic resonance
PEG	Polyethylene glycol
PAMPs	Pathogen-associated molecular patterns
POD	Peroxidase
PR	Pathogenesis-related protein
PRRs	Pattern recognition receptors
PTGS	Post-transcriptional gene silencing
PTI	PAMP-triggered immunity
RDR	RNA-dependent RNA polymerase
RISC	RNA-induced silencing complex
RLKs	Receptor-like kinases
RLCKs	Receptor-like cytoplasmic kinases
RNAi	RNA interference
ROS	Reactive oxygen species
SA	Salicylic acid
SAR	Systemic acquired resistance
siRNA	Small interfering RNA
SOD	Superoxide dismutase
SVM	Support vector machine
TCA	Tricarboxylic acid cycle
TPM	Transcripts per million
VOCs	Volatile organic compounds
WUE	Water-use efficiency
